# Men and loneliness in the Covid‐19 pandemic: Insights from an interview study with UK‐based men

**DOI:** 10.1111/hsc.13746

**Published:** 2022-02-05

**Authors:** John Ratcliffe, Mona Kanaan, Paul Galdas

**Affiliations:** ^1^ Department of Health Sciences University of York York UK

**Keywords:** coronavirus, covid‐19, loneliness, masculinity, men, SARS‐CoV‐2, social isolation

## Abstract

Since the onset of the Covid‐19 pandemic, the UK, like many countries, has had restrictions on social contact, and injunctions of ‘social distancing’. This study aimed to generate new insights into men's experiences of loneliness during the pandemic, and consider the ramifications of these for continued/future restrictions, the easing of restrictions, and the future beyond the pandemic. Twenty qualitative interviews were conducted with men between January and March 2021. A maximum variation purpose sample frame required at least three non‐white men, three LGBTQ+men, three men with a university education, three without a university education, three 18–30 years old, and three aged 60+. Thematic analysis, focused on semantic themes, was employed as part of a ‘grounded’ epistemology whereby the stated perspectives of the interviewees drove the content of the study. Seven themes were constructed: (i) lost and new activities and routines; (ii) remote social interaction; (iii) narrowed social spheres; (iv) rethought and renewed recognition of what is important; (v) loneliness with a purpose; (vi) anxiety of social contact; and (vii) easier for themselves than others. Lost routines, fewer meaningful activities, and a reduction in face‐to‐face interaction, were framed as challenges to preventing loneliness. Solo‐living gay men seemed particularly negatively affected. However, many men displayed new, more covid‐safe routines and activities. Remote forms of interaction were often utilised, and though they were imperfect, were constructed as worth engaging with, and held capacity for improvement. A moral need to reduce transmission of SARS‐COV‐2, and a fear of catching it, became important features of participants lives that also affected loneliness. Men at higher risk of health complications from Covid‐19 were particularly likely to highlight anxiety of social contact. Reducing restrictions alone may not return everyone to pre‐pandemic levels of loneliness, particularly if the pandemic remains a significant public health issue.

**What is known about this topic**

- Loneliness is a public health concern, and often a gendered experience.
- Pandemic related restrictions greatly reduced opportunities for social contact.
- We have a limited understanding of whether, and how, the pandemic influenced men's experiences of loneliness.

**What this paper adds**

- The loss of activities and routines, and a lack of face‐to‐face interaction, were significant challenges to preventing loneliness, particularly among solo‐living gay men.
- Remote interaction may be better when routinised or dependable, in smaller groups, and with a structure facilitating the opportunity to speak.
- Anxiety of Covid‐19, and a moral need to reduce transmission, were important influences on the causes and severity of loneliness.

## 1 INTRODUCTION

Loneliness has been extensively linked to poor mental and physical health (Bolton, 2012; Cacioppo et al., 2006; Schinka et al., 2012; Valtorta et al., 2016; Victor & Bowling, 2012). It is often defined as a perceived lack, or loss, of meaningful social relationships, in contrast to ‘social isolation’, which represents an objective lack of social contact (Cattan et al., 2005; Perlman & Peplau, 1981; Townsend, 1957). Cacioppo and Cacioppo (2018) posit loneliness as an evolutionary mechanism, priming individuals to seek out, and work for, mutual benefit. In this way, it remains a subjective emotion, but it is one based on actual relationships. Heylen (2010) conceptualises this by constructing two dimensions to loneliness. ‘Deficit’ loneliness is where an individual's social relationships are objectively insufficient, thus represents loneliness resulting from social isolation. ‘Cognitive’ loneliness, on the other hand, is when a person's perception of their social relationships does not meet their expectations.

For Franklin et al. (2019), this means non‐loneliness refers to a feeling of ‘belonging’. Gendered cultures, then, give rise to gendered needs, expectations, and emotional language for ‘belonging’. Connell's theory of ‘hegemonic’ masculinities (Connell, 2005; Connell and Messerschmidt, 2008) is a highly influential theoretical positioning of gendered expectations and emotional language. According to Connell, masculine ideals exist as reifications of gendered inequalities. Constructions of masculinity, therefore, often imply strength, dominance, or invulnerability, while some men, and masculinities, can be ‘subordinate’ or ‘marginalised’ (Connell, 2005). This paradigm has been cited to explain a disinclination to acknowledge or seek help for loneliness (De Jong‐Gierveld et al., 2018; Rokach, 2018). It may also frame a difficulty with forming intimate relationships (McKenzie et al., 2018; Stevens & Westerhof, 2006), that, in turn, can increase reliance on spousal relationships (Nurmi et al., 2016; McKenzie et al., 2018), or on alcohol use (Munoz‐Laboy et al., 2009). Broader cultures of family, community and work may also impact men's subjective feelings (Franklin et al., 2019). In Ratcliffe et al.’s (2021) study of older men, for example, the instrumental help of others, often in workplaces or families, could be a source of masculine ‘social worth’ and therefore less loneliness.

Since the onset of Covid‐19, several works have found evidence of aggregately increased loneliness (Killgore et al., 2020; McQuaid et al., 2021; Bu et al., 2020). McKenna‐Plumley et al. (2021) suggested this may be a result of a loss of in‐person interaction, and a loss of freedoms. Some scholars have suggested that the pandemic may have had a worse effect on women's loneliness (Jones et al., 2021; Wickens et al., 2021). However, there is a paucity of research into gendered experiences of loneliness during the pandemic. McKenna‐Plumley et al.’s (2021) study included just two men, and larger scale statistical studies of prevalence do not capture the context of the aggregate sex difference they present. If men are disinclined to acknowledge loneliness, how might that be understood and enacted in a pandemic situation in which loneliness has become a significantly greater concern? Moreover, if gendered cultures of work, family and community are central to men, how might lockdowns and injunctions of social distancing have affected practices of ‘belonging’?

The current study investigates and highlights where and how the Covid‐19 pandemic, and its accompanying social restrictions, have impacted loneliness for men, and the ramifications of this for policy and practice. The research questions were formulated as a single research question focused on men's stated experiences, and a sub‐question focused on the implications of their perspectives:

How has the Covid‐19 pandemic affected men's perceptions and experiences of loneliness?

‐What are the ramifications of these for easing restrictions, future pandemic situations, and a post‐pandemic world?

## 2 METHODS

Twenty semi‐structured interviews were conducted with men across Northern England and Scotland, between January and March 2021. A relatively ‘grounded’ approach was taken, insofar as the content of this article was strongly driven by the interviewees (Charmaz, 1996). Thematic analysis was used to analyse the data (Braun & Clarke, 2006). The study was originally designed to explore men's experiences and perceptions of loneliness more generally, but the ongoing Covid‐19 pandemic at the time of data collection meant men's accounts were frequently framed and constructed within this context.

### 2.1 Sample

To be eligible for the study, participants were not required to have experienced loneliness. A maximum variation purposive sampling frame was employed to ensure diversity (Guest et al., 2013). Masculinities are neither universal nor fixed identities (Connell, 2005), therefore a diverse sample was considered more likely to identify different experiences related to different masculinities. The sample required at least three non‐white men, three LGBTQ+men, three men with a university education, three without a university education, three 18–30 years old, and three aged 60+. Interviewees were sourced via gatekeepers in an LGBTQ+group, a sports centre, a community centre, a men's activity group, an organisation promoting good health in black people, an addiction recovery support group, and organisations supporting voluntary work. In several cases, the gatekeeper advertised the study widely, resulting in participants that were not part of the organisations contacted. Table 1 lists the demographic data of the participants.

**TABLE 1:** Demographic information of participants

Age	*N* (% of interviewees)
18–30	5 (25)
31–45	5 (25)
46–60	7 (35)
61+	3 (15)
Ethnicity	
White‐British	14 (70)
South‐Asian	4 (20)
Eastern‐European	1 (5)
White‐African	1 (5)
Sexual orientation	
Heterosexual	12 (60)
Bisexual	1 (5)
Homosexual	7 (35)
Gender orientation	
Cisgender	19 (95)
Transgender	1 (5)
Attended higher education	
Yes, in the UK	5 (25)
Yes, in another country	2 (10)
Current student	3 (15)
No	10 (50)
Living situation	
Solo‐living	8 (40)
With spouse/partner (with or without children)	7 (35)
With parents/guardians	4 (20)
With housemates	1 (5)

The study does not aim to be an exhaustive account of all men's perspectives, but to provide a selection of evidenced perspectives that may be ‘transferable’ to similar contexts. ‘Transferability’ refers to qualitative research that is applicable to other settings (Lincoln & Guba, 1985). Men constitute approximately half the world's population, therefore aiming for theoretical saturation was considered unfeasible (Low, 2019). Instead, a ‘pragmatic’ approach was taken to interviewee numbers (Braun & Clarke, 2021). Through the maximum variation purposive sampling frame, this aimed to provide suitably diverse perspectives. Once the minimum sampling criteria had been fulfilled, further interviews were no longer pursued.

### 2.2 Data collection

Interviews were conducted remotely via video call (Google hangouts, Zoom) or telephone. They lasted between 30 and 120 min, and were recorded then auto‐transcribed, or recorded on the telephone then manually transcribed. They were conducted during the third UK ‘lockdown’, between 11th January and 12th March 2021. Seventeen took place during a period where social contact was limited to members of the same household, and only ‘essential’ shops were open, and three were conducted shortly after the first stage of ‘reopening’ on 8th March, at which point only essential shops and schools were open.

The interviews followed a semi‐structured format loosely consisting of three parts. First, a less structured interview, discussing loneliness, was employed. This aimed to utilise Hollway and Jefferson’s (2000, 2008) technique of ‘free association’. This method allows participants to frame broad topics according to their own discursive associations, therefore is congruent with a ‘grounded’ epistemology. Second, participants were asked whether and how their perspectives had been impacted by the pandemic. Finally, the ‘free‐association’ method was dropped, and questions related to maleness, masculinities and loneliness were asked. This aimed to produce data able to manifest whether, and how, the men's narratives were gendered.

### 2.3 Analysis

The analysis aimed to construct ‘semantic’ themes, that is, ‘surface’ level themes portraying what the participants directly stated (Javadi & Zarea, 2016). These are fairly descriptive, so that the ensuing discussion can consider the ramifications of that which is described (Braun & Clarke, 2006). This helped to ensure the results had a clear ‘link’ to the data (Vindrola‐Padros & Johnson, 2020), assisting the employment of a ‘grounded’ epistemology. Open coding was employed, then built and narrowed into specific and consistent themes (Moghaddam, 2006). Coding was conducted in NVivo (2020), in five stages. A form of ‘decision‐trail’ (Long & Johnson, 2000), that is, a description of how the codes were formed and adapted through each stage of analysis, was created to enhance rigour. Analysis was conducted by the lead author, with the remaining authors providing feedback and validation after each stage.

1. Interviews were digitally recorded, transcribed and uploaded to Nvivo.
2. Open coding was conducted, whereby data was assigned a large number of descriptive labels broadly related to loneliness. The decision to produce an analysis focused on the pandemic was taken after this stage.
3. A second open coding was conducted, which built a large number of new codes solely related to the pandemic and loneliness.
4. Codes were reviewed, adapted and narrowed.
5. Themes were built, defined and reproduced into an article suitable format.

### 2.4 Ethics

Participants gave written consent via email. Ethics approval was granted by the ethics committee in the Department of Health Sciences, University of York. An ethical stance influenced by Plummer’s (2001) notion of ‘critical humanism’, a philosophy designed to balance individual well‐being against justice ethics, was employed. This resulted in five actions beyond basic ethical practice:

- A list of organisations able to provide help and support was provided to participants.
- An approach showing due diligence to the interviewees mental state was taken. Practically, this required sometimes asking whether the participant is OK, whether they wanted a break, or not asking potentially relevant questions if the interviewer felt it may distress the interviewee.
- Criticising the interviewee, or accusing them of unethical beliefs/actions, was avoided. However, the information sheet stated that, if serious criminal or potentially harmful behaviour was disclosed, this would be reported.
- Participants were afforded pseudonyms, and demographic data were not linked to participant pseudonyms in Table 1 (Bell, 2010; Ratcliffe et al., 2021).
- In analysis, narratives were placed within a theoretical socio‐political landscape acknowledging unequal gendered relations, and the severity of the pandemic, while retaining a view of the participant as an emotional being who had aided the study.

## 3 FINDINGS

Seven themes were constructed. ‘Lost and new activities and routines’ (3.1) summarises how the men's activities were disrupted and reformed. ‘Remote social interaction’ (3.2) describes the men's frequent tendency to critically consider remote forms of interaction. ‘Narrowed social spheres’ (3.3) notes that the men often relayed an increased focus on the home environment, and sometimes to local communities. ‘Rethought and renewed recognition of what is important’ (3.4) exemplifies how the pandemic led many to reconsider what about their lives and social connections is important. ‘Loneliness with a purpose’ (3.5) emphasises a moral imperative to prevent transmission of SARS‐COV‐2, and how that impacted their emotional experiences. ‘Anxiety of social contact’ (3.6) consisted of a fear of catching the virus, and how this impacted loneliness. Lastly, ‘easier for themselves than others’ (3.7) aimed to capture how the men often discussed other groups for whom the situation is more difficult.

### 3.1 Lost and new activities and routines

Keeping *‘busy’* was frequently cited as critical to preventing loneliness, and the pandemic was presented as a challenge to this. One man, Sam, even struggled to identify whether he was *‘lonely’* or *‘bored’*. Les placed a lot of emphasis on work, travel, and general activity, describing lockdown as *‘sitting still’*. Despite these difficulties, all of the men showed signs of adapting to their circumstances. Les did this in two ways. In the lockdown in effect at the time of the interview, he had downloaded an app on his phone that encouraged him to go running:

Les I think having a routine at the moment of some kind, has kind of saved me in that sense. because doing this every other day, the couch to 5K… I think a routine is a good thing

In this instance, the formation of a new routine was the key to ‘saving’ him, and this focus on new routines was mirrored in many of the men's accounts. Ahmad, for example, described the importance of routinely going to the park, and Alisdair the importance of evening phone calls with his brother. In the lockdown of Spring 2020, Les did not have his running app. Instead, he volunteered at a hospital, and spoke of this equally positively:

Les that was like three days, three and a half days a week. 12‐hr shifts. And, yeah, I felt like I was doing my bit, you know, just kind of involved. And socially it was good because you were, I felt like I was in the world. And you had this regular interaction with people. And it was important. And even though it wasn't like, you know, not saving lives, necessarily, but was needed. And I think that's the, the key thing … it's the feeling of being needed.

As well as being a routinised activity, this example also emphasises the meaningfulness of the activity. Again, this was mirrored in other accounts. Gary, for example, spent more time on political activism, and assisting LGBTQ+support groups, and Hassan arranged for food to be sent out to vulnerable older people via his community centre. On the other hand, many of the men humorously lamented an increase in doing mundane activities such as housework, DIY, and playing on games consoles. The pandemic, then, had led to the loss of routines, which could also lead to an uncertainty of one's social role. Much was done, though, to replace these with new roles and routines.

### 3.2 Remote social interaction

The most frequently relayed adaptation was an increased utilisation of remote forms of interaction. It was often stated to be a relatively poor substitute for what many termed *‘face‐to‐face’* interaction. A lack of physical intimacy, difficulties with understanding social context through body language, greater anxiety, a lack of equipment and/or technical ability, and difficulties with being able to get involved in conversations (because one individual would dominate), were all highlighted as problems. Some men also discussed becoming bored of it. However, most emphasised it as worth engaging with:

Jim Zoom, Google chat, etcetera, I see the good and bad sides of them. I think it's brilliant. They're brilliant connectors, brilliant ways of being able to engage with new people. Me, I’m quite a tactile person, so I like meeting people, chatting, and going through stuff face to face. But I’ve learned to adapt, to use this technology. My sons would laugh at me if, you know, I have to phone them up to ask them how to switch such and such on, I'm a real technophobe if that's the right word. But I understand the value of what we have to do, so I’ve learnt to adapt.

Perhaps in part because of this understanding of the ‘value’ of restrictions on face‐to‐face contact, some of the men also suggested ways to improve remote interactions. Smaller groups, adequate opportunity to speak and take part, and dependable and/or routinised chats, were all extolled. Saed even put forward a design for an app, which would have pre‐arranged events, with both introductions and break off groups to facilitate conversation. Remote interaction also provided some people with opportunities they had not previously had. Scott, who had a limiting physical disability, even stated that, for him, this had ‘probably’ led to more social interaction than prior to the pandemic.

### 3.3 Narrowed social spheres

Many of the men emphasised that their social spheres had narrowed into a focus on home environments. This held difficulties for some younger participants who lived with their family, such that Jonny described it a *‘pressure cooker environment’*. This was also constructed as frustrating for those seeking sexual and/or romantic partners, given that opportunities to meet people were greatly reduced. It was most openly constructed as difficult, though, by solo‐living gay men who, in this study, were more likely to have built their social connections in public spheres. Neil even contrasts this against the difficulties of those who live with families:

Neil if I want to have company, I can't really, so you know. And everyone, other people complaining about, you know, I’m fighting with my partner, or the kids are driving me up the wall and stuff, and I think, well, swap with me for a week, see what it is!

Nevertheless, for some, this narrowing of spheres facilitated stronger relationships with existing partners, family, and housemates, and several men expressed a deep gratitude for this. Some also spoke with enthusiasm for an improved ‘local community’. Broadly, then, this narrowing of spheres was a common experience which could have negative and positive effects. Living alone, though, held particular problems.

### 3.4 Rethought and renewed recognition of what is important

Nicolas summarised this by stating that the pandemic *‘made me look at my life, and who I am’*. A similar attitude was relayed by many of the men, often by noting a renewed appreciation of good aspects of life, such as good health, close relationships, economic comfort and outdoor spaces. It was also expressed as a process of introspective learning:

Neil This whole period has been really cathartic because it's allowed me to figure out what it is that does make me happy. Figure out what's good about me, figure out that I am worth enough on my own, I don't need to have somebody else to validate me.

Despite this, Neil expressed more loneliness in lockdown than most of the men. The pandemic, then, seemed able to facilitate introspective learning, yet the lockdown could impede the fulfilment of what had been learnt. Adam, for example, learnt that it was important for him to attend settings outside of his house, yet his opportunities to do so were limited. Nevertheless, the learning itself, and newfound appreciation for good aspects of life in particular, were constructed as positive developments.

### 3.5 Loneliness with a purpose

Most men suggested that they understood the rationale of the restrictions. For some, this significantly impacted their emotional experiences:

Alisdair There's a friend of mine who's on the covid ward…and you think what they're going through compared to what I'm doing, basically just sitting doing nothing, I can deal with that. Couldn't deal with what he does, but my tiny little bit of help, just to do nothing really, it's not that much to ask.

Martin described this as loneliness with a ‘purpose’, and stated there was a positive aspect to this as it gave meaning to his life. As a result, he felt particularly lonely after being invited to attend a party:

Martin If I get an invitation, which I got several times, I have to say like bloody hell don't you read a newspaper? There's another lockdown! And it's makes me feel sorry to explain to you it's inappropriate…

Interviewer Is that a kind of loneliness, in effect?

Martin Yeah. It's like a spiral down it started, and it's pushing us more and more down

Being physically alone, then, was less lonely as it represented an act of social benefit. When others failed to share this social cause, though, Martin felt lonely, even though he was being invited to spend time with other people.

### 3.6 Anxiety of social contact

Some of the participants were anxious of social contact as they were concerned about the possibility of catching Covid‐19. This was particularly salient in participants who were at higher risk of health complications:

Rhys I'm in that extremely vulnerable group because of my compromised immune system. I’m shielding up to beginning of August last year, so from March through till August you're shielding. And then you've got to keep away or you've just, you're so paranoid about going near people.

In this quote, Rhys is ‘choosing’ to avoid social contact, yet it is a choice heavily influenced by the severity of the risk to his health. Both he and other interviewees also discussed this in more emotional terms. Rhys later notes that he would feel *‘uncomfortable and vulnerable’* sitting in a restaurant, and Martin states *‘you are afraid of crowds nowadays’*. Although the anxiety is based on a specific health decision, then, it could still facilitate a lonely experience.

### 3.7 Easier for themselves than others

Most of the men believed they found the situation easier than others did. Some believed the restrictions may be more difficult for younger people because, as Martin put it, it is a time where people *‘develop within a social group’*. However, the younger participants in this study did not identify this. Strikingly, participants with mental health problems often believed this an advantage, as it prepared them for the situation:

Jim People who suddenly couldn't have what they always had couldn't get their heads around why they couldn't have it anymore. But I was already on that journey before because I lost all of that before I got into my (alcohol addiction) recovery.

Hassan believed people he described as ‘BME’ tend to receive more attention from their children, reducing their loneliness during the pandemic. South Asian interviewees in this study all spoke of regular and intimate social contact with children and/or parents, often because they lived in fluid multi‐generational housing. Ahmad and Faisal even stated that they were not lonely because of this, although they did not relate it to the pandemic. Hassan also believed ‘BME’ people tended to be less trusting of services, thus may be less likely to receive pandemic‐related assistance. Again, this did not feature specifically in other interviews, but Faisal was critical of support services, particularly care homes, in such a way that it resonated with Hassan's perception.

## 4 DISCUSSION

This study explored how the Covid‐19 pandemic affected men's perceptions and experiences loneliness. Findings demonstrated that restrictions could result in loneliness, but this only told part of the story. During restrictions, a loss of activities, and the loss of face‐to‐face interactions, were particularly felt. Nevertheless, new activities and routines, a sense of local community, and a clear understanding of the ‘purpose’ of the restrictions, that was understood and respected by others, could do much to alleviate loneliness. Remote forms of communication were imperfect, but they could be positive, and held capacity for improvement. Anxiety of catching Covid‐19, and changes to routines in relation to that, meant loneliness could result from a fear of the pandemic. Men who are not young, South Asian men, and men who had experienced severe mental health problems, relayed reasons they experienced less loneliness than others. Solo‐living gay men, and men with pre‐existing health conditions that placed them at additional risk from Covid‐19, sometimes showed greater pandemic‐related loneliness.

Ratcliffe et al. (2021) posited that older men may place a sense of ‘social worth’ as critical to preventing loneliness, and emphasise that this does not always require social contact. Though their study refers solely to older men, ‘loneliness with a purpose’ similarly placed avoiding social contact as an act of social benefit, thus able to reduce loneliness. It may therefore represent a stark example of the importance of ‘social worth’ to men's loneliness. Indeed, Kamin et al.’s (2021) study of solo‐living women in Slovenia related a similar moral responsibility to reduce transmission, but did *not* suggest this reduced loneliness.

Participants’ social spheres were narrowed onto the home environment, and those who were married all expressed a thankfulness for their spousal relationship. Many pre‐pandemic studies suggest men's loneliness is more affected by the existence of a spouse than women's (Bergland et al., 2016; Nowland et al., 2018; Pinquart & Sorensen, 2001), therefore these narrowed spheres may offer additional explanation for research suggesting women have been more negatively affected by the pandemic (Wicken et al., 2021; Jones et al., 2021). This may also help explain why solo‐living men identifying as gay reported greater difficulties than other solo‐living men. Domesticity has been treated critically as heteronormative by many LGBTQ+commentators, in favour of a queer public identities (Gorman‐Murray, 2020). For solo‐living gay men, then, restrictions may have undermined a more outside of the home focused social environment.

In contrast to this study, research from Mind (2020) and Gillard et al. (2021) found that people with mental health problems faced additional psychological difficulties during the pandemic. Bartholomaeus and Tarrant (2016) suggest that older men may construct a masculine identity as a ‘sage’, a man who has experience and knowledge of the world such that they negotiate it more effectively. Though this study again refers to older men, the tendency for the men to place the pandemic as ‘easier for themselves than others’ may represent a masculine discourse in which a ‘sage’ is able to protect themselves from loneliness. It may even resonate with the ‘rethought and renewed recognition of what is important’, in that this may represent the construction of an identity as a ‘sage’. A tendency to downplay personal experiences of loneliness, in favour of constructing an identity as a ‘sage’, would be consistent with work suggesting men understate mental health concerns (Rokach, 2018; Yousaf et al., 2015). Nevertheless, some of the men who had experienced past loneliness did appear to possess a genuine resilience, indicating that it could provide tools to overcome loneliness once more.

Gillard et al. (2021) also suggested that people from ethnic minorities have faced additional mental health challenges, primarily due to racism exacerbated by the pandemic. The South Asian men in this study, though, posited extended family environments as a benefit in comparison to other ethnic groups. This emphasises the different dimensions of people's experiences, but more research is required to understand the impact of the pandemic on loneliness in men from different ethnic groups. Men with pre‐existing health conditions, and older men, are known to be at higher risk of health complications from Covid‐19 (Wolff et al., 2021; Chen et al., 2020). This study found that these men sometimes experienced particular anxiety of social contact, which rendered them more likely to experience loneliness, although this remained present in some less ‘at risk’ men. Time, vaccines, and lower case rates, as well as more covid‐safe social interactions, may alleviate this anxiety.

As in previous infectious disease pandemics, Covid‐19 appeared to have destabilised social structures (Cava et al., 2005; Strong, 1990), and this was a key element of the ‘lost activities and routines’. However, Covid‐19 has lasted longer than the periods addressed by Strong (1990) and Cava et al. (2005), perhaps explaining why this study found more signs of new routines and behaviours. The emphasis placed on meaningful activities may display a masculine practice, given that these often focused on helpful tasks (Franklin et al., 2019; Ratcliffe et al., 2021). New routines, along with rethought recognition of what is important, and social spheres narrowed over an extended period, may result in smaller, but closer, social networks for years, particularly if people remain anxious of social contact.

### 4.1 Study limitations

Constructing semantic themes may limit insight (Braun & Clarke, 2006). The original interview schedule was not designed to generate evidence specifically about the Covid‐19 pandemic, potentially limiting the depth and richness of data. In particular, socio‐economic status did not feature in this paper as it was not semantically related to both loneliness and the pandemic. This study cannot gauge the scale of these themes across societies (Bryman, 2016). The sample was fairly diverse, yet no‐one was black, a single parent, or either under 20 or over 71 years old. Recruitment of the sample came via support groups and community groups, therefore these men may be more community orientated than average, and with greater access to social support. No participant had experienced Covid‐19, and only one participant mentioned a person they knew who had. As such, the study offers limited insight to people with experience of the virus, particularly bereaved people who may be at risk of loneliness (Stroebe and Schut, 2020). Time with family in multi‐generational households, and involvement in local communities, were constructed as beneficial, yet involve social contact that may increase transmission. It is impossible to derive from this data whether, when, and to what extent, anxiety of Covid‐19 is a rational response, or a cognitive problem.

This study was conducted with men, but few other studies examine this topic, rendering it difficult to ascertain whether, and how, these findings are gendered. Some work has found parallel results without claiming them to be gendered. Kremers et al. (2021) conducted a qualitative study of older people in the Netherlands, and found that people stated they were less lonely because they understood the purpose of restrictions. Statistical work has found that increases in loneliness do not return to pre‐covid levels during periods of no restrictions (Killgore et al., 2020). The results of this study, which often emphasise the pandemic as a potential pathway to loneliness, rather than restrictions per se, may offer some explanation for this. It is necessary to place the findings of this study as constructed by men, and with masculine features, but which may not be specific to men.

### 4.2 Implications for policy and practice

While restrictions could constitute a pathway to loneliness, conceptualising the problem as ‘restrictions equal loneliness’ was insufficient. The men were aware of the health risks posed by Covid‐19, and this impacted their emotional needs. During times where restrictions are being eased, it may be important to balance anxieties, and new routines, against the preference for ‘face‐to‐face’ interaction. This may be further complicated by a ‘fear of missing out’ (Baker et al., 2016), such that people may feel a pressure to return to face‐to‐face settings. An emphasis on ‘personal responsibility’ (Williams, 2021) may be difficult for some, given a complex backdrop of anxiety, and a notion of ‘loneliness with a purpose’. Community services may need to take covid‐cautious approaches, and communicate with people in a manner acknowledging the possibility of these anxieties and/or moral perspectives.

During severe restrictions, the loss of face‐to‐face interaction was frequently cited as difficult, and solo‐living gay men may be particularly prone to loneliness. This suggests support for allowing ‘support bubbles’ (HM government, 2021), i.e., a named person or household with who someone who lives alone can spend physical time with, albeit this may need to be balanced against public health risks. For services wishing to utilise remote forms of interaction, it is notable that smaller groups, where people felt involved and able to speak, and were dependable in terms of their availability, were constructed as better. The benefits of routinised activities suggests that clear and consistent government rules and advice, with less frequent changes, may be beneficial to preventing loneliness. Having safe, meaningful, and routinised activities appeared to be the ultimate arbiters of the men's loneliness during the Covid‐19 pandemic.

## AUTHORSHIP CONTRIBUTION

John Ratcliffe contributed to conceptualisation; data curation; formal analysis; investigation; methodology; visualisation; writing—original draft; and project administration; Mona Kanaan contributed to supervision; validation; and writing—review & editing; Paul Galdas contributed to conceptualisation; methodology; supervision; validation; and writing—review & editing.

## CONFLICTS OF INTEREST

The authors declare no conflicts of interest.

## Data Availability

Data not shared. Sharing data compromises ethical standards. Decision trail and interview schedule are available on request. The authors thank everyone who took part in an interview, and everyone who assisted in finding men to interview.
